# SpecBEV: An End-to-End BEV 3D Object Detection Algorithm Based on Frequency-Domain Analysis and Geometric Alignment

**DOI:** 10.3390/s26113551

**Published:** 2026-06-03

**Authors:** Yu Lin, Shijie Jia

**Affiliations:** School of Electrical Engineering, Dalian Jiaotong University, Dalian 116028, China; 20232303@djtu.edu.cn

**Keywords:** BEV object detection, multi-view fusion, frequency prior, spatial attention, cross-view feature alignment

## Abstract

This paper proposes SpecBEV, an enhanced multi-view 3D object detection framework for autonomous driving using bird’s-eye-view (BEV) representations. Compared with LiDAR-based methods, multi-camera perception offers higher cost-effectiveness and flexibility. However, existing end-to-end BEV detectors suffer from illumination variations, occlusions, and cross-view inconsistencies during feature projection and fusion. These issues often introduce redundant background activations and geometric misalignment in the BEV space, leading to missed detections, false positives, and unstable localization. To address them, we introduce a frequency-prior spatial attention module (SA-Freq). It utilizes fixed discrete cosine transform (DCT) bases to model the multi-band responses of BEV features and produce spatial attention weights that suppress redundant activations and enhance target-related regions. We further design a cross-view feature alignment module (CFA) to ensure consistency between single-view BEV features and the fused BEV representation, thereby reducing geometric inconsistency and improving localization stability. Experiments on the nuScenes validation set demonstrate that SpecBEV achieves 0.3856 in mAP and 0.4871 in NDS. Compared with the BEVDet baseline, it yields an absolute gain of 0.1028 (36.35% relative improvement) in mAP and an absolute gain of 0.1371 (39.17% relative improvement) in NDS, which validates the effectiveness of the proposed method.

## 1. Introduction

In recent years, multi-view camera-based 3D object detection has attracted increasing attention in autonomous driving perception. Compared with LiDAR-based 3D detection methods, multi-camera vision-based perception offers several advantages, including lower cost, more flexible deployment, and wider coverage, and has thus emerged as a pivotal research direction in autonomous driving perception [1,2,3,4]. The bird’s-eye-view (BEV) representation projects multi-view image features into a unified shared space, which facilitates spatial geometric modeling and cross-view information fusion. As a result, it has become an important research paradigm in vision-based 3D object detec-tion [5,6,7,8,9].

However, existing end-to-end multi-view BEV 3D object detection methods still suffer from two key challenges during cross-view feature projection and fusion [10,11,12]. First, multi-view inputs typically contain substantial repetitive or irrelevant background information. After being mapped into the BEV space, these redundant responses may interfere with the representation of target regions, weaken the discriminative capability of target-related features, and consequently degrade detection accuracy [13,14]. Second, owing to variations in camera positions, viewing angles, and projection paths, the fused BEV features may still suffer from geometric deviations and semantic misalignment, which can further lead to missed detections, false positives, and unstable localization and orientation estimation [15,16,17,18,19,20].

Existing studies on multi-view visual BEV representation learning can generally be grouped into two main technical routes: methods based on explicit depth modeling or geometric lifting, and methods based on Transformer- or query-based implicit spatial modeling [1,2,3]. In addition, some studies attempt to directly learn the mapping from perspective views to the BEV space through purely network-based structures [21]. Nevertheless, most existing methods mainly focus on the construction of BEV representations [22,23], while lacking targeted designs for suppressing redundant background responses in fused features and enforcing cross-view geometric consistency constraints [15,16,17,18].

To address these issues, this paper proposes an improved framework for end-to-end multi-view BEV 3D object detection, termed SpecBEV. The proposed method improves the quality of BEV representations from two aspects, namely feature enhancement and cross-view alignment. Specifically, a frequency-prior spatial attention module (SA-Freq) is designed to model the multi-band responses of BEV features using fixed discrete cosine transform (DCT) [24] frequency bases and to generate spatial attention weights, thereby suppressing redundant background responses while enhancing the representation of target-related regions [25,26,27,28]. In addition, a cross-view consistency constraint (CFA) is introduced to enforce consistency between per-view BEV features and the fused BEV representation, thereby alleviating cross-view geometric inconsistency and improving the stability of object localization and orientation estimation.

The main contributions of this paper are summarized as follows: (1) A frequency-prior spatial attention module, SA-Freq, is proposed to introduce frequency-domain modeling into multi-view BEV representation learning, thereby suppressing redundant background responses and enhancing target-region feature representation. (2) A cross-view feature alignment, CFA, is proposed to alleviate geometric deviations in multi-view fusion and improve localization stability by enforcing consistency between per-view BEV features and the fused BEV representation. (3) Experimental results on the nuScenes dataset demonstrate that the proposed method effectively improves the performance of multi-view vision-based 3D object detection.

## 2. Related Work

### 2.1. Vision-Based BEV 3D Object Detection

Vision-based 3D object detection has become an important research direction in autonomous driving perception. Compared with 2D object detection, 3D detection is required not only to recognize object categories, but also to estimate 3D attributes such as spatial location, size, and orientation. However, achieving accurate 3D object detection solely from camera inputs remains highly challenging, mainly because the 2D imaging process cannot naturally preserve the depth and spatial geometric information of real-world scenes. In recent years, with the development of BEV perception, 3D object detection based on camera-only BEV representation has attracted increasing attention [1,2,3,5,6,7,8,9,10,11,12]. Existing surveys categorize camera-only 3D perception into three primary components, namely 2D feature extraction, view transformation, and 3D decoding, among which the view transformation module is the key to establishing the mapping from perspective images to a unified BEV representation.

With regard to the construction of representations from perspective views to the BEV space, existing methods have mainly evolved along two mainstream technical routes [1,2,3]. One route is based on explicit depth modeling or geometric lifting, represented by methods such as LSS, BEVDet, and BEVDepth [5,6,7]. These methods typically predict pixel-wise depth distributions, lift image features into 3D space, and then compress and aggregate them onto the BEV plane. As a result, they exhibit strong geometric interpretability and have significantly promoted the development of multi-view vision-based 3D detection [5,6,7]. The other route is based on 3D-to-2D projection or Transformer/query mechanisms, represented by methods such as OFT, BEVFormer, PETR, and DETR3D [9,10,11,12,29]. These methods usually employ positional encoding, 3D reference points, or cross-view attention mechanisms to implicitly model spatial information, thereby enhancing global contextual representation and cross-view feature interaction [10,11,12,29,30]. In addition to these two mainstream routes, some studies attempt to directly learn the mapping from perspective views to the BEV space through MLPs or purely network-based structures, such as M^2^BEV [21], in order to reduce the reliance on explicit geometric modeling. Although these methods have achieved remarkable progress in BEV representation construction, explicit depth-based methods are usually sensitive to the accuracy of depth estimation and projection [7,8,31,32,33], whereas implicit modeling methods often incur high computational cost and still exhibit certain limitations in modeling long-range objects and fine-grained spatial relationships when explicit geometric constraints are absent.

Overall, existing vision-based BEV 3D object detection methods mainly focus on how to construct representations from image views to the BEV space, and have achieved substantial progress in depth estimation, view transformation, and cross-view feature interaction. However, the further optimization of the fused BEV representation itself remains insufficient. On the one hand, most existing methods [13,14,15,16,17,18,19,20] emphasize the projection and fusion process, while lacking dedicated mechanisms to suppress redundant background responses in fused features. On the other hand, due to the inherent differences in camera positions, viewing angles, and projection paths, the fused BEV features may still suffer from geometric deviations and semantic misalignment, thereby affecting the stability and localization accuracy of object detection.

### 2.2. Frequency-Domain Methods and Feature Enhancement

Frequency-domain analysis has been widely applied in image processing and visual representation learning [34,35,36,37,38,39]. Unlike methods that model features only in the spatial domain, frequency-domain approaches characterize input signals from the perspective of frequency distribution, thereby providing complementary information for feature enhancement in terms of global structures and local details [36,37]. In recent years, with the development of deep learning, frequency-domain modeling has gradually been introduced into neural networks [38]. Existing studies can generally be divided into two categories. One category transforms features from the spatial domain to the frequency domain for direct modeling, where Fourier, Wavelet, and other transforms are employed to capture responses in different frequency bands [34,38,39]. The other category embeds frequency-domain information into the network to facilitate attention modeling or local feature enhancement, for example, by introducing discrete cosine transform (DCT) [24] into attention mechanisms to improve the perception of multi-frequency information [26]. Existing studies have shown that frequency-domain modeling can enhance responses to informative components to a certain extent and reduce the interference of redundant components in feature learning.

However, existing frequency-domain methods have mainly been applied to image classification, image reconstruction, image enhancement, and general visual representation tasks, while their application to multi-view BEV representation learning remains relatively limited. In particular, in BEV 3D object detection, fused BEV features usually contain repeated background responses, irrelevant region activations, and accumulated cross-view projection errors [13,14,15,16,17,18]. How to further enhance the discriminative capability of target-related regions and suppress redundant background responses by exploiting frequency priors remains insufficiently explored. In fused BEV representation optimization, frequency-domain modeling is expected to highlight target-related responses and suppress unnecessary background interference, leading to more discriminative feature learning for multi-view BEV 3D object detection.

### 2.3. Cross-View Consistency and Geometric Alignment

The key to multi-view visual perception lies in the effective alignment and fusion of information from different views [40,41]. Due to the inherent differences in camera installation positions, viewing angles, and imaging paths, the same object often exhibits different appearance characteristics and spatial relationships across different views, which brings challenges to unified BEV representation learning. To mitigate the influence of view discrepancies, existing multi-view detection methods [17,18,19,29,40] usually perform feature alignment and fusion through explicit geometric projection, depth estimation, or cross-view attention interaction, thereby improving the integration capability of multi-view information to a certain extent.

However, relying solely on feature projection and fusion operations cannot fully guarantee the consistency of multi-view features in the BEV space. Especially under occlusion, illumination variation, and complex background interference, features from different views may still suffer from geometric deviations and semantic misalignment after being mapped into a unified space, thereby affecting the stability of the fused representation. Existing studies have shown that consistency learning and alignment constraints can effectively improve feature alignment accuracy and representation robustness in multi-view tasks [40,41]. Nevertheless, in multi-view BEV 3D object detection, explicit modeling of the consistency relationship between per-view BEV features and the fused global BEV representation remains relatively limited. Therefore, introducing cross-view consistency constraints during the fusion process is of great importance for alleviating geometric inconsistency and improving the stability of object localization and orientation estimation.

## 3. Method

### 3.1. Overview

**Figure 1:**
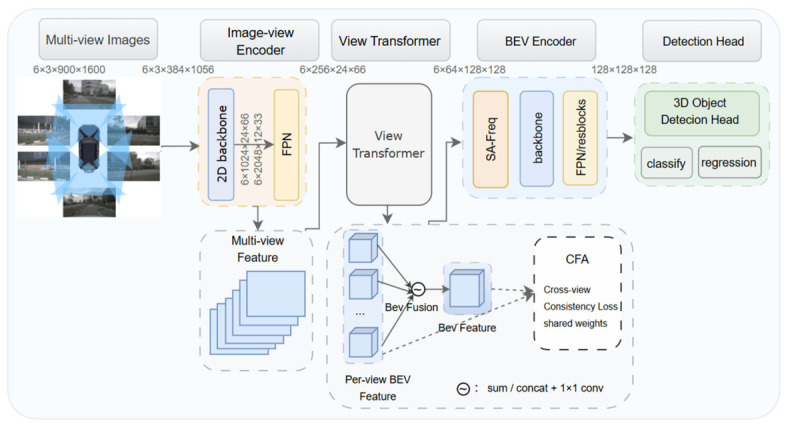
Overall framework of SpecBEV.

This section presents SpecBEV, an enhanced framework for end-to-end multi-view 3D object detection, which aims to boost detection performance by generating higher-quality BEV representations. The proposed framework mainly consists of an image feature encoder, a view transformation module, a BEV feature optimization module, and a detection head, as illustrated in Figure 1. Specifically, the image feature encoder is used to extract multi-scale semantic features from multi-view input images, the view transformation module projects image-domain features into a unified BEV space, the BEV feature optimization module further enhances and constrains the fused representation, and the detection head performs final object classification and 3D bounding box regression.

Specifically, the input consists of synchronized image sequences captured by a surrounding six-camera system. First, images from all views are processed by a shared-weight CNN [42] backbone to extract image features, which are then fed into a feature pyramid network [43] to obtain multi-scale semantic representations. The features at different scales are subsequently compressed to a fixed channel dimension to match the input requirement of the subsequent view transformation module. Under the current setting, the six input images are resized to 1056 × 384; the backbone and FPN produce a unified image-domain feature of 6 × 256 × 24 × 66, which is then projected by the view transformation module to generate per-view BEV features of 6 × 64 × 128 × 128. In this work, ResNet-101-DCN [42] is adopted as the backbone network, and the model is initialized with pretrained FCOS3D [44] parameters to enhance feature extraction capability and accelerate convergence.

After the image-domain features are obtained, the proposed method projects the image features from each camera view into a unified BEV space through the view transformation module. Considering the differences in camera position and orientation, this module performs geometric projection based on the intrinsic and extrinsic camera parameters, thereby achieving spatial coordinate alignment and semantic aggregation. Rather than performing direct cross-view fusion, the proposed framework initially generates an independent per-view BEV representation for each camera so as to preserve, as much as possible, the geometric and semantic differences among different views in BEV space.

**Figure 2:**
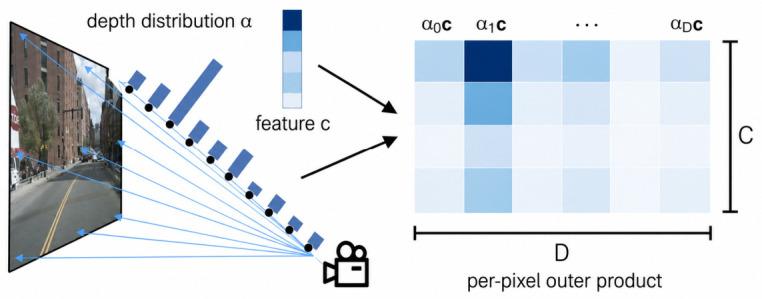
View transformation module based on the lift-splat paradigm [5]. The image feature of each pixel is lifted along the predicted depth distribution to form frustum features, which are then projected and pooled into the BEV space. The colors are used for visual distinction only and do not indicate quantitative meanings.

Specifically, the view transformation module follows the lift-splat paradigm adopted in LSS [5] to accomplish the mapping from image space to BEV space, as illustrated in Figure 2. For each camera view, the network first predicts a discrete depth distribution for each pixel location based on the image features, and then expands the 2D image features along the depth dimension to construct a frustum feature representation. Subsequently, with the aid of the intrinsic and extrinsic camera parameters, the frustum features are transformed from the image coordinate system to the ego-vehicle coordinate system and projected onto the predefined BEV grid according to their spatial locations. Finally, the features at different depths are accumulated onto the bird’s-eye-view plane through voxel aggregation or BEV pooling, yielding the corresponding per-view BEV representation. In this way, the 2D semantic information in the image domain can be explicitly mapped into a unified 3D space, thereby providing the basis for subsequent multi-view fusion and BEV representation optimization.

The input images from different views are first transformed into per-view BEV representations through the view transformation module, and are then fused to obtain the global BEV representation. On this basis, SA-Freq is employed during the BEV encoding stage for feature enhancement, while CFA is introduced during training to impose consistency supervision between the per-view BEV representations and the fused global BEV representation.

After the view features are transformed into the unified BEV space, the obtained BEV features are further refined in the feature optimization stage. To address the redundant background responses and cross-view geometric inconsistency that commonly arise after multi-view fusion, the proposed method improves the BEV representation from two aspects, namely feature enhancement and consistency regularization. Specifically, a frequency-prior spatial attention module (SA-Freq) is introduced during the BEV feature encoding stage. This module models the multi-band responses of BEV features using fixed discrete cosine transform (DCT) [24] frequency bases and generates spatial attention weights [25,26,27], thereby suppressing redundant background responses while enhancing target-related regions. Meanwhile, a cross-view feature alignment (CFA) is introduced during training to enforce consistency between the per-view BEV features generated by each view and the fused global BEV representation. In this way, the geometric deviations introduced during multi-view projection and fusion can be alleviated, and the spatial consistency and semantic stability of the fused representation can be improved.

The refined BEV representations are subsequently delivered to the detection head to perform 3D object detection. This head is composed of two components, namely a classification branch for category prediction and a regression branch for estimating the position, scale, and orientation of each target. Through the collaboration of the above components, SpecBEV is able to effectively improve the discriminative capability and geometric alignment quality of multi-view BEV representations while preserving the end-to-end property of the overall framework.

During training, the proposed method adopts joint optimization of the main detection loss and the cross-view consistency loss, while the detailed definition of the consistency constraint is provided in Section 3.3.

### 3.2. Frequency-Prior Spatial Attention Module (SA-Freq)

In multi-view BEV 3D object detection, although cross-view features can be projected and fused into a unified spatial representation, this process often introduces a large amount of redundant background responses. In particular, under occlusion, illumination variation, or complex scene interference, the fused BEV features usually contain both background components irrelevant to the detection task and valid responses related to target objects. Such redundant information not only weakens the discriminability of target regions, but also affects the ability of the subsequent detection head to model spatial context, thereby degrading the overall detection accuracy. To address this issue, a frequency-prior spatial attention module (SA-Freq) is designed. This module employs fixed discrete cosine transform (DCT) bases to model frequency responses and generate spatial attention weights to highlight target-related regions while suppressing redundant background responses.

**Figure 3:**
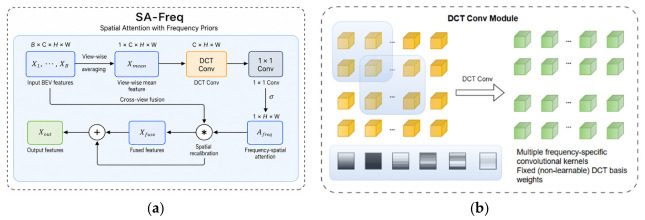
Illustration of the SA-Freq module. (**a**) Overall structure of SA-Freq; (**b**) Implementation of DCT-based convolution using fixed frequency bases. In the figure, “+” denotes element-wise addition, and “*” denotes element-wise multiplication.

The SA-Freq module is embedded at the input of the BEV Encoder, and its input is the per-view BEV feature generated in the view transformation stage, denoted as $X^{’}\in R^{B\times C\times H\times W}$, where B denotes the number of views, C denotes the channel dimension, and H×W denotes the spatial resolution in BEV space. Inside the module, frequency-guided spatial weights are first generated by aggregating features along the view dimension, and these weights are then used to recalibrate the fused global BEV features. The enhanced features are subsequently fed into the following BEV encoder. The overall structure is shown in Figure 3a.

Specifically, considering that the BEV features generated from different views exhibit strong similarity in overall spatial frequency structure, the proposed method first computes mean aggregation along the view dimension to obtain an intermediate representation $X_{mean}$:

(1) $$X_{mean}=\frac{1}{B}\sum_{b=1}^{B}X_{b},\;{\;X}_{b}\in R^{C\times H\times W},$$

where $X_{b}$ denotes the per-view BEV feature corresponding to the b-th view. This view aggregation operation is only used to generate frequency-domain attention weights and does not replace the final cross-view feature fusion. Through this operation, redundant inter-view variations can be reduced while preserving the spatial distribution characteristics in BEV space, thereby making subsequent frequency-spectrum modeling more stable.

To clarify the implementation of the frequency projection, the 2D DCT basis can be written as

(2) $$B_{u,v}^{i,j}=cos\left(\frac{\pi u}{H}\left(i+\frac{1}{2}\right)\right)cos\left(\frac{\pi v}{W}\left(j+\frac{1}{2}\right)\right),$$

where (u,v) denotes the frequency index and (i,j) denotes the spatial position. Accordingly, the projection of a spatial feature $X\in R^{H\times W}$ onto the (u,v) frequency basis is defined as

(3) $$f_{u,v}=\sum_{i=0}^{H-1}\sum_{j=0}^{W-1}X_{i,j}B_{u,v}^{i,j}.$$

This formulation shows that the response under each frequency basis can be expressed as a weighted summation over the spatial feature map, which can be naturally implemented in convolutional form using fixed DCT basis kernels.

After obtaining $X_{mean}$, a 2D DCT convolution kernel is introduced to perform frequency-domain mapping and generate the frequency feature tensor:

(4) $$F_{freq}={\mathrm{Conv}}_{\mathrm{DCT}}\left(X_{mean}\right),{\;F}_{freq}\in R^{K\times H\times W},$$

where K denotes the number of selected frequency bases. Each selected basis corresponds to one predefined 2D DCT frequency component, and the associated response map measures the activation strength of the intermediate BEV representation under that frequency pattern. In the present implementation, these selected components are determined according to the size of the adopted DCT kernel, so that different kernel sizes correspond to different values of *K*. In implementation, the selected DCT bases are instantiated as fixed, non-learnable convolution kernels. Therefore, ${Conv}_{DCT}$ is not a conventional learnable spatial convolution, but an explicit frequency projection operator that maps $X_{mean}$ into K response maps under different predefined frequency components. Since these kernels are preconstructed from orthogonal DCT bases and remain fixed during training, the frequency projection itself introduces no additional learnable parameters; only the subsequent lightweight mapping is trainable.

Compared with conventional spatial-domain compression or direct spatial convolution, this explicit multi-band decomposition provides a more structured basis for attention generation. Conventional averaging operations mainly preserve coarse global statistics and are closely related to low-frequency responses, which is often insufficient to describe the complete frequency structure of the feature. In contrast, the proposed DCT-based projection decomposes the intermediate BEV representation into multiple frequency bands before attention generation. Among them, low-frequency responses mainly reflect the coarse spatial layout and large-scale structural distribution in BEV space, whereas relatively higher-frequency responses are more sensitive to local variations, object boundaries, and fine-grained target-related patterns. This enables the module to distinguish structured target-related responses from redundant background activations more effectively.

Based on the frequency feature $F_{freq}$, a 1×1 convolution is further adopted to linearly combine the frequency channels, followed by a Sigmoid activation function to generate the spatial attention map $A_{freq}$:

(5) $$A_{freq}\;=\;\sigma \left({Conv}_{1\times 1}\left(F_{freq}\right)\right),\;A_{\mathrm{freq}}\;\in \;R^{1\times H\times \;W},$$

where ${Conv}_{1\times 1}$ is used to compress and map the frequency dimension, and σ(⋅) denotes the Sigmoid function. The resulting $A_{freq}$ represents the enhancement or suppression coefficients at different positions in BEV space. When multiplied with the fused feature, it is broadcast along the channel dimension according to the spatial positions. This attention map reflects the saliency differences in BEV regions in the frequency domain.

After the frequency-guided spatial attention map is obtained, the per-view BEV features from all views are first fused to produce the global BEV representation $X_{fuse}$:

(6) $$X_{fuse}=Fuse\left(X_{1},X_{2}\ldots X_{B}\right),X_{fuse}\in R^{C\times H\times W},$$

where Fuse(⋅) denotes the cross-view feature fusion operation. Since the view-averaged feature can provide a more stable estimate of the spatial frequency structure, whereas the cross-view fused feature contains more complete semantic information, the former is used to generate the frequency-guided spatial weights, while the latter is explicitly recalibrated. The spatial attention map $A_{freq}$ is then used to recalibrate the fused feature position by position, and a residual connection is adopted to maintain feature stability. The enhanced output feature is formulated as follows:

(7) $$X_{out}=X_{fuse}+X_{fuse}⊙A_{freq},$$

where ⊙ denotes element-wise multiplication. The attention map $A_{freq}$ acts on the fused feature in the spatial dimension and is broadcast along the channel dimension. This residual enhancement strategy enables explicit recalibration of the fused BEV features without modifying the backbone structure. For target-related regions, the module enhances their response intensity, whereas for irrelevant background regions, it suppresses redundant activations.

From the perspective of the operating mechanism, SA-Freq first performs explicit multi-band frequency projection on the intermediate BEV representation and then generates spatial attention weights based on the resulting frequency responses. In this way, the module combines frequency-domain analysis with spatial recalibration, thereby suppressing redundant background activations, strengthening target-related regions, and improving the discriminability of fused BEV features for the subsequent detection head.

### 3.3. Cross-View Feature Alignment (CFA)

In multi-view BEV 3D object detection, different cameras naturally differ in imaging position, viewpoint direction, and projection path. Even after unified view transformation, the BEV features generated from different views may still exhibit geometric deviations and semantic misalignment in the spatial domain. Such cross-view inconsistency may accumulate during subsequent feature fusion, thereby affecting the stability of the fused representation and further leading to localization drift, unstable orientation estimation, and degraded detection accuracy. To alleviate this issue, a cross-view feature alignment (CFA) is introduced at the end of the encoder. By enforcing consistency between the BEV features generated from each view and the fused global BEV representation, the alignment of multi-view features in the shared space can be strengthened, thereby alleviating geometric errors introduced during multi-view fusion.

Unlike methods that perform alignment directly in the image domain, CFA is imposed in the BEV space. The reason is that the BEV features after view transformation have already been mapped into a unified spatial coordinate system, which can more directly reflect the consistency of different views in terms of spatial geometry and semantic representation. Specifically, assume that there are N camera views. After the View Transformer and BEV Encoder, the per-view BEV feature generated from the i-th view is denoted as $F_{BEV}^{i}\in R^{C\times H\times W}$, while the fused global BEV feature is denoted as $F_{BEV}^{fused}\in R^{C\times H\times W}$. The fused representation is taken as the alignment reference, guiding all view-specific branches to gradually converge to a consistent spatial representation during training. Although the fused BEV representation may still contain residual errors introduced during multi-view projection and fusion, it provides more complete global spatial context than any single-view BEV feature alone. Therefore, CFA is used only as an auxiliary consistency regularization during training, rather than a hard constraint that replaces or overwrites the original view-specific representations.

Specifically, the proposed method employs the pixel-wise $L_{2}$ norm to measure the discrepancy between the BEV feature from a single view and the fused BEV feature. The alignment errors from all views are then averaged to define the cross-view consistency loss as

(8) $$L_{cfa}=\frac{1}{N}\sum_{i=1}^{N}{∥F_{BEV}^{i}-F_{BEV}^{fused}∥}_{2}^{2},$$

where N denotes the total number of camera views, and $∥\cdot {∥}_{2}^{2}$ represents the pixel-wise squared $L_{2}$ distance. Taking the fused global BEV feature as the consistency target, this loss constrains the local BEV representation generated from each view to remain consistent with the global feature in terms of spatial distribution and semantic expression. Different from relying solely on feature fusion itself, such explicit consistency supervision can directly reduce the representational discrepancy among different views during training, thereby mitigating the instability caused by the accumulation of cross-view errors.

During optimization, CFA serves as an auxiliary regularization term and is jointly trained with the main detection loss. The overall optimization objective consists of the detection loss and the cross-view consistency loss, and is formulated as

(9) $$L=\left(1-\lambda \right)L_{det}+\lambda L_{cfa},$$

where $L_{det}$ denotes the classification and regression loss of the detection head, $L_{cfa}$ denotes the cross-view consistency loss, and λ is a hyperparameter used to balance the two loss terms. In this work, λ is set to 0.5 by default. Since this constraint is only introduced during training, it does not introduce additional computational overhead during inference. This design enables the framework to further improve the stability and robustness of multi-view fused representations while maintaining detection efficiency.

By introducing explicit consistency supervision in the shared BEV space, CFA can effectively improve the geometric alignment accuracy of multi-view features, reduce feature shifts caused by viewpoint differences, and enhance the stability of the fused representation. The resulting consistency-aware BEV feature provides a more reliable spatial representation for the subsequent detection head, which is beneficial for improving the robustness of object localization and orientation estimation.

## 4. Experiments

This section first introduces the dataset, evaluation metrics, and implementation details used in this study. Then, the proposed SpecBEV is compared with existing state-of-the-art multi-view BEV 3D object detection methods on the nuScenes [45] validation set. Finally, ablation studies and qualitative analysis are conducted to further verify the effectiveness of the two key components, namely SA-Freq and CFA.

### 4.1. Dataset and Evaluation Metrics

**Figure 4:**
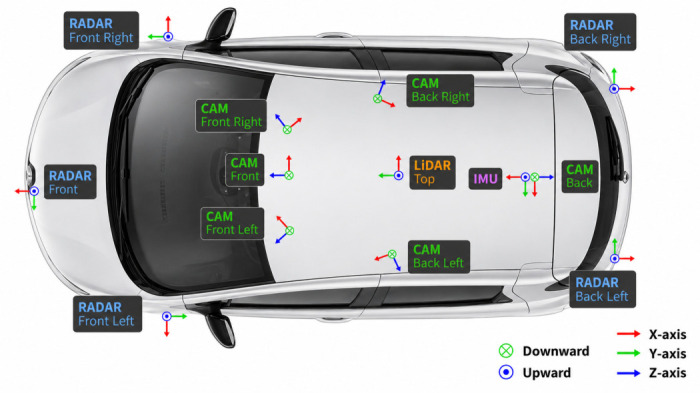
Sensor configuration of the data acquisition platform [45].

The proposed SpecBEV is evaluated on the nuScenes dataset. nuScenes is a widely used large-scale multi-sensor benchmark in autonomous driving, containing synchronized observations collected from six cameras, five millimeter-wave radars, and one LiDAR sensor, thereby providing 360° environmental coverage around the ego vehicle. The data acquisition platform and sensor configuration are illustrated in Figure 4. The dataset contains diverse urban road scenes under various challenging environmental conditions, making it suitable for systematically evaluating the robustness and generalization capability of multi-view 3D object detection methods in real traffic scenarios [45].

The nuScenes dataset contains 1000 scenes in total, including 700 scenes for training, 150 scenes for validation, and 150 scenes for testing. Each scene lasts approximately 20 s, and keyframes are sampled at a fixed frequency. The resolution of each image is 1600 × 900, and all objects in each scene are annotated with complete 3D bounding box information, including position, size, and orientation. In this work, RGB images from the six surround-view cameras are used as input. After image feature extraction and view transformation, the features are projected into a unified BEV space. Under the current setting, the projected per-view BEV representation is constructed on a 128 × 128 BEV grid.

To comprehensively evaluate the detection performance on the nuScenes dataset, the official evaluation protocol is adopted, including mean Average Precision (mAP) and nuScenes Detection Score (NDS). Among them, mAP is used to measure the detection accuracy of the model under different object categories and distance thresholds. Under the nuScenes evaluation protocol, mAP is computed based on the two-dimensional center distance between the predicted box and the ground-truth box, and the average precision of each category is calculated under multiple distance thresholds. It is defined as

(10) $$mAP=\frac{1}{|C||D|}\sum_{c\in C}\sum_{d\in D}AP(c,d),$$

where C  denotes the set of object categories, corresponding to the 10 detection classes in nuScenes, and D denotes the set of distance thresholds, defined as {0.5,1,2,4} m. Here, AP(c,d) represents the average precision computed for category c at distance threshold d.

Building upon this, NDS combines detection accuracy with several true positive error measures, thereby providing a more comprehensive evaluation of the overall performance of 3D object detection models. This metric reflects not only whether a target is correctly detected, but also the errors in object localization, scale estimation, orientation prediction, and velocity estimation. It is defined as

(11) $$NDS=\frac{1}{10}[5\cdot mAP+\sum_{mTP\in M}\left(1-min(1,mTP)\right)],$$

where M denotes the set of five true positive error metrics, including mean Average Translation Error (mATE), mean Average Scale Error (mASE), mean Average Orientation Error (mAOE), mean Average Velocity Error (mAVE), and mean Average Attribute Error (mAAE).

In addition to mAP and NDS, the above five official nuScenes true positive error metrics are also reported [45]. Specifically, mATE measures the translation error of the object center, mASE evaluates the scale discrepancy between the predicted box and the ground-truth box, mAOE reflects the orientation estimation error, mAVE measures the velocity prediction error, and mAAE evaluates the attribute prediction error. Since lower values of these metrics indicate better detection quality, they are jointly reported with mAP and NDS in the subsequent experiments to provide a more comprehensive analysis of the detection performance of SpecBEV.

### 4.2. Implementation Details

All experiments were conducted on an NVIDIA GeForce RTX 4060 Laptop GPU with 8 GB of memory (Nvidia Corporation, Santa Clara, CA, USA). The software environment was based on CUDA 12.4 and PyTorch 1.13. To ensure the stability and reproducibility of the experimental process, both training and evaluation were performed on a single GPU.

In our implementation, the source images with a resolution of 1600 × 900 are resized and cropped to 1056 × 384 before being fed into the image backbone. For network implementation, ResNet-101-DCN [42] was adopted as the image feature extraction backbone, and the model was initialized with pretrained FCOS3D [44] parameters to enhance feature extraction capability and accelerate convergence. The input consists of synchronized image sequences captured by six surround-view cameras. Image features are first extracted by a shared-weight CNN backbone and then fed into a feature pyramid network to obtain multi-scale semantic representations. The resulting features are subsequently compressed to a fixed channel dimension to match the subsequent view transformation and BEV representation learning stages.

During training, the AdamW [46] optimizer was employed, with the initial learning rate set to 2 × 10^−4^. A cosine annealing strategy was adopted for learning rate decay. The model was trained for 120 epochs, and evaluation was conducted on the nuScenes validation set after each epoch. The total batch size for the six-camera input was set to 6. In addition, synchronized batch normalization was adopted to improve training stability. During training, standard data augmentation strategies, including random rotation, scaling, and flipping, were applied to increase the diversity of the training samples.

**Figure 5:**
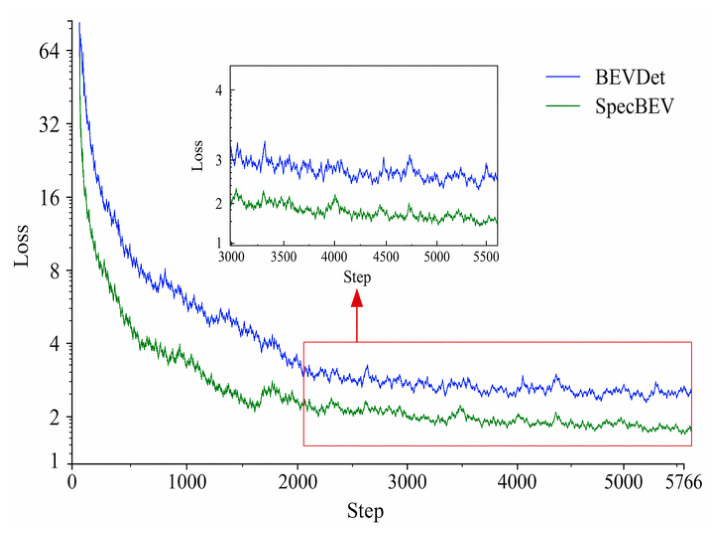
Training loss curves of BEVDet and Ours.

To further illustrate the optimization behavior of the proposed method during training, the loss curves of the BEVDet baseline and SpecBEV are depicted in Figure 5. Notably, both methods converge gradually as training proceeds. Compared with the baseline, SpecBEV maintains a lower loss level and exhibits more stable convergence in the later training stage. The zoomed-in view further shows that the proposed method achieves consistently lower loss values after convergence, which indicates that the introduced modules help improve the training effectiveness and optimization stability of the overall framework.

### 4.3. Main Results

#### 4.3.1. Comparison with State-of-the-Art Methods

The proposed SpecBEV was quantitatively compared with several mainstream multi-view BEV 3D object detection methods on the nuScenes validation set, including BEVDet [6], BEVDet4D [22], BEVDepth [7], and FastBEV [23], and the results are presented in Table 1.

From Table 1, it can be observed that SpecBEV achieves the best overall detection performance among the compared methods. Specifically, SpecBEV achieves 0.3856 in mAP and 0.4871 in NDS on the validation set, both of which are superior to those of the other methods. Compared with the baseline BEVDet [6], SpecBEV improves mAP from 0.2828 to 0.3856, corresponding to an absolute gain of 0.1028 and a relative improvement of 36.35%. Meanwhile, NDS increases from 0.3500 to 0.4871, corresponding to an absolute gain of 0.1371 and a relative improvement of 39.17%. These results indicate that the proposed method can effectively improve the quality of multi-view BEV representations and further enhance the overall detection performance.

In addition to the primary metrics, SpecBEV also shows better performance on several true positive error metrics. In particular, mATE, mASE, and mAOE are all significantly reduced compared with the baseline, indicating that the proposed method provides more stable performance in object localization, scale estimation, and orientation prediction. These results demonstrate that, while preserving the end-to-end detection framework, SpecBEV can achieve better 3D object detection performance by improving the discriminability of the fused BEV representation and enhancing cross-view consistency.

To further evaluate the practical efficiency of the proposed method, we additionally compare SpecBEV with the BEVDet baseline in terms of parameter count, computational cost, and inference speed under different input resolutions. The corresponding results are summarized in Table 2.

As shown in Table 2, SpecBEV introduces only a limited increase in parameter count and computational cost compared with the BEVDet baseline, while achieving clear improvements in detection accuracy under both input resolutions. Specifically, under the input resolution of 704 × 256, the parameter count increases from 54.1 M to 54.5 M, and the computational cost increases from 223.6 GFLOPs to 229.0 GFLOPs, while mAP and NDS improve from 0.2693 and 0.3426 to 0.3441 and 0.4410, respectively. Under the input resolution of 1056 × 384, although the computational cost increases from 452.0 GFLOPs to 463.5 GFLOPs and the inference speed decreases slightly from 9.3 FPS to 8.9 FPS, the proposed method still achieves clear gains in mAP and NDS, reaching 0.3856 and 0.4871, respectively. These results indicate that SpecBEV attains a favorable trade-off between detection performance and computational efficiency.

#### 4.3.2. Visualization Results

**Figure 6:**
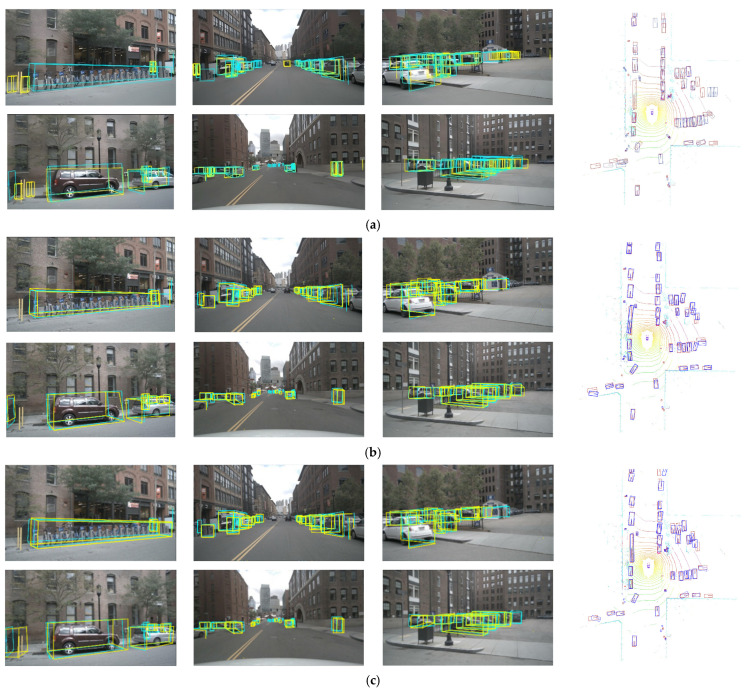
Qualitative comparison of detection results among BEVDet, FastBEV, and SpecBEV in the first representative scene. In each subfigure, the (**left**) side shows the six-camera-view detection results, where the yellow boxes denote the predicted boxes and the blue boxes denote the ground-truth boxes. The (**right**) side shows the corresponding BEV visualization, where the dark blue boxes denote the predicted boxes and the red boxes denote the ground-truth boxes. (**a**) BEVDet; (**b**) FastBEV; (**c**) Ours.

**Figure 7:**
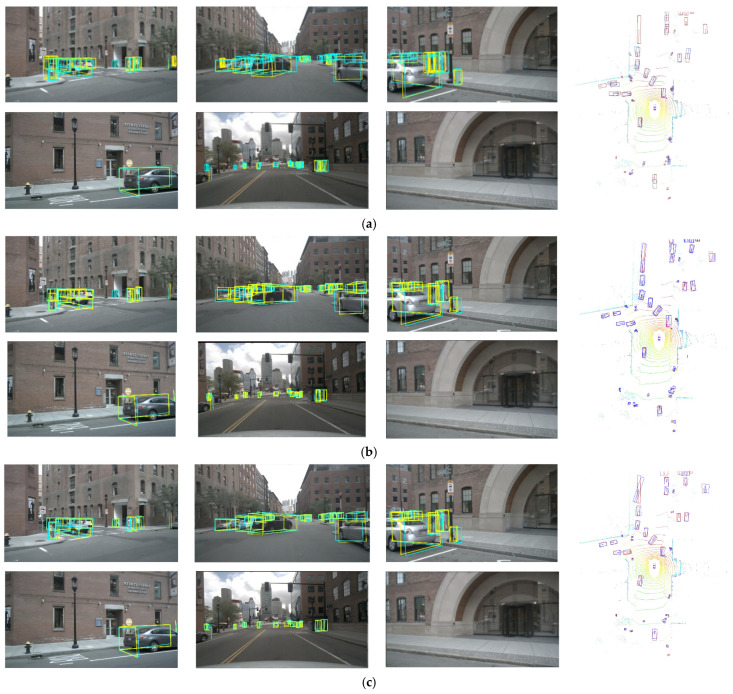
Qualitative comparison of detection results among BEVDet, FastBEV, and SpecBEV in the second representative scene. In each subfigure, the (**left**) side shows the six-camera-view detection results, where the yellow boxes denote the predicted boxes and the blue boxes denote the ground-truth boxes. The (**right**) side shows the corresponding BEV visualization, where the dark blue boxes denote the predicted boxes and the red boxes denote the ground-truth boxes. (**a**) BEVDet; (**b**) FastBEV; (**c**) Ours.

To further evaluate the effectiveness of the proposed method in complex driving scenes, qualitative comparisons were conducted among BEVDet, FastBEV, and SpecBEV, and the corresponding results are shown in Figure 6 and Figure 7.

In each figure, the left side of each subfigure presents the detection results from the six camera views, while the right side presents the corresponding bird’s-eye-view (BEV) visualization. For the six-view images, the yellow boxes denote the predicted boxes and the blue boxes denote the ground-truth boxes. For the BEV visualization, the dark blue boxes denote the predicted boxes and the red boxes denote the ground-truth boxes.

As shown in Figure 6, clear differences can be observed among the three methods in the first representative scene. BEVDet produces relatively coarse detection results in several regions, and some predicted boxes exhibit deviations in spatial position and orientation. FastBEV improves the overall scene perception to some extent, but inaccurate localization and incomplete predictions can still be observed for several objects, especially in regions with dense targets or complex background structures. In comparison, SpecBEV produces prediction results that are more consistent with the ground-truth annotations in both the image views and the BEV space. The predicted boxes are more concentrated around the target regions, and their positions and orientations are more stable, indicating that the proposed method can provide a more reliable fused BEV representation in complex multi-view scenes.

Figure 7 further presents the qualitative comparison in another representative scene. In this scene, the differences between the compared methods are more clearly reflected in object completeness, localization consistency, and spatial structure preservation. BEVDet shows missed or unstable predictions for several targets, and some predicted boxes in the BEV space deviate noticeably from the ground-truth layout. FastBEV alleviates some of these issues, but prediction instability can still be observed in certain distant or densely distributed target regions. By contrast, SpecBEV achieves more complete detections and more accurate geometric alignment with the ground truth. In both the six-view results and the BEV visualization, the predicted boxes of SpecBEV better match the spatial distribution of real targets, demonstrating that the proposed method is more effective in suppressing redundant interference and improving cross-view representation consistency.

### 4.4. Ablation Studies

#### 4.4.1. Module-Wise Ablation Study

To verify the contribution and effectiveness of each component in SpecBEV, a series of ablation experiments were conducted on the nuScenes validation set. All experiments were performed based on BEVDet [6], where different modules and their combinations were progressively introduced to analyze their independent contributions and collaborative effects on the overall performance. In addition to the baseline setting and the single-module variants, the complete model was further evaluated under different values of the balance coefficient λ  in CFA. The corresponding results are summarized in Table 3.

In Table 3, M denotes the baseline model without SA-Freq and CFA, A denotes the configuration with CFA only, and B denotes the configuration with SA-Freq only. The rows denoted as C(λ) represent the complete model equipped with both SA-Freq and CFA under different values of the balance coefficient λ. The performance of these configurations is reported using mAP, NDS, and five true positive error metrics (mATE, mASE, mAOE, mAVE, and mAAE), so as to evaluate not only the individual gains and complementarity of the proposed modules, but also the influence of the balance coefficient in the complete model.

Both modules bring stable performance improvements. When only CFA is introduced, the model is improved from M to A, where mAP increases from 0.2828 to 0.3208, and NDS increases from 0.3500 to 0.4008. At the same time, mATE and mAOE decrease from 0.7734 and 0.6976 to 0.6805 and 0.6087, respectively. These results indicate that the cross-view consistency constraint can effectively alleviate geometric alignment errors in multi-view fusion and improve the stability of object localization and orientation estimation.

When only SA-Freq is introduced, the model is improved from M to B, where mAP increases to 0.3451 and NDS increases to 0.4268. Meanwhile, mATE, mASE, and mAOE are further reduced. Compared with the configuration using CFA only, SA-Freq yields more evident gains in the primary metrics, indicating that the frequency-prior spatial attention mechanism can effectively suppress redundant background responses in the fused BEV representation and enhance the feature representation of target-related regions, thereby improving the overall detection accuracy.

When both SA-Freq and CFA are introduced, the complete model consistently outperforms the baseline, while its performance varies with the balance coefficient λ. As λ increases from 0.3 to 0.5, both mAP and NDS improve steadily, indicating that an appropriate consistency constraint is beneficial for reducing cross-view inconsistency and enhancing the quality of the fused BEV representation. Among the tested settings, the complete model with λ=0.5  achieves the best overall performance, with mAP = 0.3856, NDS = 0.4871, mATE = 0.5970, mASE = 0.2355, mAOE = 0.5523, mAVE = 0.4581, and mAAE = 0.2136. When λ  is further increased to 0.6, the overall detection performance drops noticeably, suggesting that excessively emphasizing the auxiliary consistency regularization may interfere with the optimization of the main detection objective. These results show that SA-Freq and CFA exhibit good complementarity in feature enhancement and cross-view alignment, while λ=0.5  provides the most balanced trade-off between the main detection task and the auxiliary consistency constraint.

#### 4.4.2. Analysis of the Frequency-Selection Setting in SA-Freq

To further analyze the influence of the frequency-selection setting in SA-Freq, additional experiments were conducted by varying the kernel size of the DCT-based frequency projection while keeping the other settings unchanged. Different kernel sizes correspond to different numbers of selected frequency components, denoted by K, and therefore control the amount of frequency information involved in attention generation. The corresponding results are reported in Table 4.

As shown in Table 4, the detection performance of SpecBEV varies with the frequency-selection setting in SA-Freq. When the kernel size is relatively small, the corresponding frequency projection is insufficient to capture diverse frequency responses in the BEV feature, which limits the effectiveness of the generated spatial attention. As the kernel size increases from 3×3 to 7×7, both mAP and NDS consistently improve, indicating that richer multi-band frequency information is beneficial for distinguishing target-related responses from redundant background activations. Meanwhile, all the reported error metrics are gradually reduced as the kernel size increases, showing that a larger frequency-selection range is also beneficial for improving localization, scale estimation, orientation prediction, velocity estimation, and attribute prediction. Among the tested settings, the 7×7 kernel achieves the best overall performance. Therefore, the 7×7 DCT kernel, corresponding to K=49, is adopted in the final model.

## 5. Conclusions

This paper proposes an improved method for end-to-end multi-view BEV 3D object detection, termed SpecBEV. Built upon the BEVDet framework, the proposed method introduces a frequency-prior spatial attention module (SA-Freq) and a cross-view consistency constraint (CFA) to optimize the fused BEV representation from two aspects, namely feature enhancement and cross-view alignment. Specifically, SA-Freq models the multi-band responses of BEV features using fixed DCT frequency bases and generates spatial attention weights to suppress redundant background responses while enhancing target-related regions. In contrast, CFA imposes a consistency constraint between the per-view BEV representations and the fused global BEV representation, thereby alleviating the geometric inconsistency introduced during multi-view projection and fusion. Experimental results show that SpecBEV achieves 0.3856 mAP and 0.4871 NDS on the nuScenes validation set, which represents a clear improvement over the baseline BEVDet, with 0.2828 mAP and 0.3500 NDS.

Further experimental analysis verifies the effectiveness of each component in the proposed method. The ablation study shows that introducing CFA alone improves the baseline performance to 0.3208 mAP and 0.4008 NDS, while also significantly reducing key error terms such as mATE and mAOE. When SA-Freq and CFA are jointly introduced, the complete model achieves the best results in terms of mAP, NDS, and multiple true positive error metrics, demonstrating their good complementarity in feature enhancement and cross-view alignment. Qualitative results further show that SpecBEV can reduce missed detections and false positives in challenging scenarios with frequent occlusions and dense traffic participants, while producing predicted boxes whose positions and orientations are more consistent with the true geometric layout. These results indicate that the proposed method exhibits good robustness and generalization capability in complex road environments.

Although the proposed method achieves competitive performance in multi-view BEV 3D object detection, there is still room for further investigation. At present, the experiments are primarily carried out on the nuScenes validation set, and the generalization ability of the proposed approach remains to be verified on more datasets and in more complex real-world scenarios. In particular, for challenging cases such as nighttime low-illumination conditions, severe occlusions, dense traffic participants, and distant small objects, how to further improve the robustness of multi-view fused representations and the reliability of detection remains an important problem. In addition, although the proposed method introduces only limited additional computational overhead during inference, extra optimization constraints are still involved during training. Therefore, future work may further explore more efficient training strategies to better balance detection accuracy and practical deployment efficiency.

## Figures and Tables

**Table 1:** Quantitative comparison of SpecBEV with state-of-the-art methods on the nuScenes validation set. ↑ indicates that higher values are better, whereas ↓ indicates that lower values are better. In the VT column, “√” indicates that the method adopts an LSS-style depth-based view transformation, and “--” indicates that the method does not use this type of view transformation. Bold values indicate the best results for each metric.

ID	Year	VT	mAP ↑	NDS ↑	mATE ↓	mASE ↓	mAOE ↓	mAVE ↓	mAAE ↓
BEVDet	2022	√	0.2828	0.3500	0.7734	0.2884	0.6976	0.8637	0.2908
BEVDet4D	2022	√	0.3235	0.4241	0.6884	0.2723	0.6732	0.4590	0.2842
BEVDepth	2023	√	0.3441	0.4410	0.7280	0.2783	0.5561	0.5131	0.2355
FastBEV	2024	--	0.3288	0.4590	0.6455	0.2922	**0.4570**	**0.4250**	0.2343
Ours	--	√	**0.3856**	**0.4871**	**0.5970**	**0.2355**	0.5523	0.4581	**0.2136**

**Table 2:** Comparison of parameter count, computational cost, inference speed, and detection accuracy between SpecBEV and the BEVDet baseline under different input resolutions. ↑ indicates that higher values are better. Bold values indicate the best detection accuracy under the corresponding input resolution.

Method	Input Resolution	Params (M)	GFLOPs	FPS	mAP ↑	NDS ↑
BEVDet	704 × 256	54.1	223.6	14.3	0.2693	0.3426
BEVDet	1056 × 384	54.1	452.0	9.3	0.2828	0.3500
Ours	704 × 256	54.5	229.0	13.6	0.3441	0.4410
Ours	1056 × 384	54.5	463.5	8.9	**0.3856**	**0.4871**

**Table 3:** Ablation study of different module configurations and balance coefficient settings. ↑ indicates that higher values are better, whereas ↓ indicates that lower values are better. “√” indicates that the corresponding module is used, and “--” indicates that the corresponding module is not used. Bold values indicate the best results for each metric.

ID	SAF	CFA	mAP ↑	NDS ↑	mATE ↓	mASE ↓	mAOE ↓	mAVE ↓	mAAE ↓
M	--	--	0.2828	0.3500	0.7734	0.2884	0.6976	0.8637	0.2908
A	--	√	0.3208	0.4008	0.6805	0.2622	0.6087	0.7825	0.2621
B	√	--	0.3451	0.4268	0.6489	0.2530	0.5701	0.7356	0.2495
C (λ=0.3)	√	√	0.3414	0.4319	0.7151	0.2797	0.5786	0.5165	0.2421
C (λ=0.4)	√	√	0.3641	0.4644	0.6283	0.2495	0.5626	0.4742	0.2378
C (λ=0.5)	√	√	**0.3856**	**0.4871**	**0.5970**	**0.2355**	0.5523	0.4581	**0.2136**
C (λ=0.6)	√	√	0.3374	0.4229	0.6355	0.2722	**0.4570**	**0.4250**	0.2343

**Table 4:** Sensitivity analysis of the frequency-selection setting in SA-Freq. Different kernel sizes correspond to different numbers of selected frequency components K. ↑ indicates that higher values are better, whereas ↓ indicates that lower values are better. Bold values indicate the best results for each metric.

Kernel Size	K	mAP ↑	NDS ↑	mATE ↓	mASE ↓	mAOE ↓	mAVE ↓	mAAE ↓
3 × 3	9	0.3724	0.4716	0.6241	0.2607	0.5596	0.4725	0.2287
5 × 5	25	0.3781	0.4781	0.6018	0.2516	0.5642	0.4677	0.2241
7 × 7	49	**0.3856**	**0.4871**	**0.5970**	**0.2355**	**0.5523**	**0.4581**	**0.2136**

## Data Availability

The data supporting the findings of this study are available from the nuScenes dataset at https://www.nuscenes.org/ (accessed on 10 May 2026).

## Abbreviations

Abbreviations	Full Term	Mathematical Formulation/Definition
BEV	Bird’s-Eye View	
CNNs	Convolutional Neural Networks	
DCT	Discrete Cosine Transform	
conv	Convolutional Operation	$conv(i,j)=\sum_{m=0}^{k_{h}-1}\sum_{n=0}^{k_{w}-1}I(i+m,\;j+n)\cdot K(m,n)+b$ I: Input feature map K: $k_{h}\times k_{w}$ convolution kernel B: Bias term
mAP	mean Average Precision	$mAP=\frac{1}{|C||D|}\sum_{c\in C}\sum_{d\in D}AP(c,d)$
NDS	NuScenes Detection Score	$NDS=\frac{1}{10}\left[5\cdot mAP+\sum_{mTP\in M}\left(1-min(1,mTP)\right)\right]$, M={mATE,mASE,mAOE,mAVE,mAAE}
mATE	Mean Average Translation Error	$mATE=1-\frac{1}{N}\sum_{i=1}^{N}{∥t_{i}^{pred}-t_{i}^{gt}∥}_{2}$, $t^{pred}$: Predicted bounding box center coordinates $\left(x,y,z\right)$. $t^{gt}$: Ground truth bounding box center coordinates.
mASE	Mean Average Scale Error	$mASE=1-\frac{1}{N}\sum_{i=1}^{N}IoU\;\left(B_{i}^{pred},\;B_{i}^{gt}\right)$, $B^{pred}$: Predicted bounding box dimensions (length, width, height). $B^{gt}$: Ground truth bounding box dimensions.
mAOE	Mean Average Orientation Error	$mAOE=\frac{1}{N}\sum_{i=1}^{N}|\theta_{i}^{pred}-\theta_{i}^{gt}|(\theta \in [-\pi ,\pi ])$, $\theta^{pred}$: Predicted yaw angle. $\theta^{gt}$: Ground truth yaw angle.
mAVE	Mean Average Velocity Error	$mAVE=1-\frac{1}{N}\sum_{i=1}^{N}{∥v_{i}^{pred}-v_{i}^{gt}∥}_{2}$, $v^{pred}$: Predicted velocity vector. $v^{gt}$: Ground truth velocity.
mAAE	Mean Average Attribute Error	$mAAE=\frac{1}{N}\sum_{i=1}^{N}II\;\left(a_{i}^{pred}\neq a_{i}^{gt}\right)$, $a^{pred}$: Predicted attribute $a^{gt}$: Ground truth attribute II(): Indicator function

## References

[1] Li H., Sima C., Dai J., Wang W., Lu L., Wang H., Zeng J., Li Z., Yang J., Deng H. (2024). Delving into the devils of bird’s-eye-view perception: A review, evaluation and recipe. IEEE Trans. Pattern Anal. Mach. Intell..

[2] Li H., Zhao Y., Zhong J., Wang B., Sun C., Sun F. (2025). Delving into the secrets of BEV 3D object detection in autonomous driving: A comprehensive survey. IEEE Trans. Intell. Transp. Syst..

[3] Ma Y., Wang T., Bai X., Yang H., Hou Y., Wang Y., Qiao Y., Yang R., Zhu X. (2024). Vision-centric bev perception: A survey. IEEE Trans. Pattern Anal. Mach. Intell..

[4] Huang K., Shi B., Li X., Li X., Huang S., Li Y. (2022). Multi-modal sensor fusion for auto driving perception: A survey. arXiv.

[5] Philion J., Fidler S. (2020). Lift, splat, shoot: Encoding images from arbitrary camera rigs by implicitly unprojecting to 3D. Computer Vision–ECCV 2020: 16th European Conference, Glasgow, UK, 23–28 August 2020, Proceedings, Part XIV 16.

[6] Huang J., Huang G., Zhu Z., Ye Y., Du D. (2021). BEVDet: High-performance multi-camera 3D object detection in bird-eye-view. arXiv.

[7] Li Y., Ge Z., Yu G., Yang J., Wang Z., Shi Y., Sun J., Li Z. (2023). BEVDepth: Acquisition of reliable depth for multi-view 3D object detection. Proc. AAAI Conf. Artif. Intell..

[8] Reading C., Harakeh A., Chae J., Waslander S.L. (2021). Categorical depth distribution network for monocular 3d object detection. Proceedings of the 2021 IEEE/CVF Conference on Computer Vision and Pattern Recognition (CVPR).

[9] Roddick T., Kendall A., Cipolla R. (2018). Orthographic feature transform for monocular 3d object detection. arXiv.

[10] Li Z., Wang W., Li H., Xie E., Sima C., Lu T., Qiao Y., Dai J. (2022). BEVFormer: Learning bird’s-eye- view representation from multi-camera images via spatio temporal transformers. European Conference on Computer Vision.

[11] Liu Y., Wang T., Zhang X., Sun J. (2022). Petr: Position embedding transformation for multi-view 3d object detection. European Conference on Computer Vision.

[12] Wang Y., Guizilini V.C., Zhang T., Wang Y., Zhao H., Solomon J. (2022). Detr3d: 3d object detection from multi-view images via 3d-to-2d queries. Conference on Robot Learning.

[13] Zhu Z., Zhang Y., Chen H., Dong Y., Zhao S., Ding W., Zhong J., Zheng S. (2023). Understanding the robustness of 3D object detection with bird’s-eye-view representations in autonomous driving. Proceedings of the 2023 IEEE/CVF Conference on Computer Vision and Pattern Recognition (CVPR).

[14] Wang S., Zhao X., Xu H.M., Chen Z., Yu D., Chang J., Yang Z., Zhao F. (2023). Towards domain generalization for multi-view 3D object detection in bird-eye-view. Proceedings of the 2023 IEEE/CVF Conference on Computer Vision and Pattern Recognition (CVPR).

[15] Song Z., Yang L., Xu S., Liu L., Xu D., Jia C., Jia F., Wang L. (2024). Graphbev: Towards robust bev feature alignment for multi-modal 3d object detection. European Conference on Computer Vision.

[16] Borse S., Klingner M., Kumar V.R., Cai H., Almuzairee A., Yogamani S., Porikli F. (2023). X-align: Cross-modal cross-view alignment for bird’s-eye-view segmentation. Proceedings of the IEEE/CVF Winter Conference on Applications of Computer Vision.

[17] Tian P., Wang Z., Cheng P., Wang Y., Wang Z., Zhao L., Yan M., Yang X., Sun X. (2024). Ucdnet: Multi-uav collaborative 3d object detection network by reliable feature mapping. IEEE Trans. Geosci. Remote Sens..

[18] Shi P., Zhou M., Dong X., Yang A. (2024). Att-BEVFusion: An Object Detection Algorithm for Camera and LiDAR Fusion Under BEV Features. World Electr. Veh. J..

[19] Pan B., Sun J., Leung H.Y.T., Andonian A., Zhou B. (2020). Cross-view semantic segmentation for sensing surroundings. IEEE Robot. Autom. Lett..

[20] Zhou B., Krähenbühl P. (2022). Cross-view transformers for real-time map-view semantic segmentation. Proceedings of the 2022 IEEE/CVF Conference on Computer Vision and Pattern Recognition (CVPR).

[21] Xie E., Yu Z., Zhou D., Philion J., Anandkumar A., Fidler S., Luo P., Alvarez J.M. (2022). M^2^BEV: Multi-camera joint 3D detection and segmentation with unified birds-eye view representation. arXiv.

[22] Huang J., Huang G. (2022). Bevdet4d: Exploit temporal cues in multi-camera 3d object detection. arXiv.

[23] Li Y., Huang B., Chen Z., Cui Y., Liang F., Shen M., Liu F., Xie E., Sheng L., Ouyang W. (2024). Fast-BEV: A fast and strong bird’s-eye-view perception baseline. IEEE Trans. Pattern Anal. Mach. Intell..

[24] Ahmed N., Natarajan T., Rao K.R. (1974). Discrete cosine transform. IEEE Trans. Comput..

[25] Qin Z., Zhang P., Wu F., Li X. (2021). Fcanet: Frequency channel attention networks. Proceedings of the 2021 IEEE/CVF International Conference on Computer Vision (ICCV).

[26] Yu K., Zhang T., Wang H., Xu Q. (2025). FSTA-SNN: Frequency-Based Spatial-Temporal Attention Module for Spiking Neural Networks. Proc. AAAI Conf. Artif. Intell..

[27] Garg I., Chowdhury S.S., Roy K. (2021). Dct-snn: Using dct to distribute spatial information over time for low-latency spiking neural networks. Proceedings of the 2021 IEEE/CVF International Conference on Computer Vision (ICCV).

[28] Hu J., Shen L., Sun G. (2018). Squeeze-and-excitation networks. Proceedings of the 2018 IEEE/CVF Conference on Computer Vision and Pattern Recognition.

[29] Jiang Y., Zhang L., Miao Z., Zhu X., Gao J., Hu W., Jiang Y.-G. (2023). Polarformer: Multi-camera 3d object detection with polar transformer. Proc. AAAI Conf. Artif. Intell..

[30] Vaswani A., Shazeer N., Parmar N., Uszkoreit J., Jones L., Gomez A.N., Kaiser Ł., Polosukhin I. (2017). Attention is all you need. Advances in Neural Information Processing Systems 30.

[31] Peng L., Wu X., Yang Z., Liu H., Cai D. (2022). Did-m3d: Decoupling instance depth for monocular 3d object detection. European Conference on Computer Vision.

[32] Chen Y., Liu S., Shen X., Jia J. (2020). Dsgn: Deep stereo geometry network for 3d object detection. Proceedings of the 2020 IEEE/CVF Conference on Computer Vision and Pattern Recognition (CVPR).

[33] Rukhovich D., Vorontsova A., Konushin A. (2022). Imvoxelnet: Image to voxels projection for monocular and multi-view general-purpose 3d object detection. Proceedings of the 2022 IEEE/CVF Winter Conference on Applications of Computer Vision (WACV).

[34] Brigham E.O. (1988). The Fast Fourier Transform and Its Applications.

[35] Burrus C.S. (2015). Wavelets and Wavelet Transforms.

[36] Huang Z., Zhang Z., Lan C., Zha Z.-J., Lu Y., Guo B. (2023). Adaptive frequency filters as efficient global token mixers. Proceedings of the 2023 IEEE/CVF International Conference on Computer Vision (ICCV).

[37] Patro B.N., Namboodiri V.P., Agneeswaran V.S. (2025). Spectformer: Frequency and attention is what you need in a vision transformer. 2025 IEEE/CVF Winter Conference on Applications of Computer Vision (WACV).

[38] Guo S., Yong H., Zhang X., Ma J., Zhang L. (2023). Spatial-frequency attention for image denoising. arXiv.

[39] Kong L., Dong J., Ge J., Li M., Pan J. (2023). Efficient frequency domain-based transformers for high-quality image deblurring. Proceedings of the 2023 IEEE/CVF Conference on Computer Vision and Pattern Recognition (CVPR).

[40] Li Q., Wang Y., Wang Y., Wang Y., Zhao H. (2022). Hdmapnet: An online hd map construction and evaluation framework. 2022 International Conference on Robotics and Automation (ICRA).

[41] Saha A., Mendez O., Russell C., Bowden R. (2022). Translating images into maps. 2022 International Conference on Robotics and Automation (ICRA).

[42] He K., Zhang X., Ren S., Sun J. (2016). Deep residual learning for image recognition. Proceedings of the 2016 IEEE Conference on Computer Vision and Pattern Recognition (CVPR).

[43] Lin T.Y., Dollár P., Girshick R., He K., Hariharan B., Belongie S. (2017). Feature pyramid networks for object detection. Proceedings of the 2017 IEEE Conference on Computer Vision and Pattern Recognition (CVPR).

[44] Wang T., Zhu X., Pang J., Lin D. (2021). Fcos3d: Fully convolutional one-stage monocular 3d object detection. Proceedings of the 2021 IEEE/CVF International Conference on Computer Vision Workshops (ICCVW).

[45] Caesar H., Bankiti V., Lang A.H., Vora S., Liong V.E., Xu Q., Krishnan A., Pan Y., Baldan G., Beijbom O. (2020). nuScenes: A multimodal dataset for autonomous driving. Proceedings of the 2020 IEEE/CVF Conference on Computer Vision and Pattern Recognition (CVPR).

[46] Loshchilov I., Hutter F. (2017). Decoupled weight decay regularization. arXiv.

